# Nonsurgical management of a tricuspid valvular pedunculated papillary fibroelastoma

**DOI:** 10.1186/1476-7120-7-44

**Published:** 2009-09-04

**Authors:** Sang-Hoon Seol, Dong-Soo Kim, Yang-Chun Han, Ki-Hun Kim, Young-Bok Kim, Dong-Kie Kim, Ung-Kim, Tae-Hyun Yang, Dae-Kyeong Kim, Doo-Il Kim

**Affiliations:** 1 Department of Medicine, Inje University College of Medicine, Busan Paik Hospital, Busan, Korea

## Abstract

A 25-year-old woman with a history of kidney transplantation for lupus nephritis was referred for the evaluation and management of a mass incidentally found on echocardiography. An oval and pedunculated mass attached to the tricuspid valve was managed with nonsurgical treatment. No symptoms and complications attributable to the mass developed. Three years later, the size of the mass decreased. Here we report the case of a probable cardiac papillary fibroelastoma (PFE), a mobile mass, with a stalk on the septal leaflet of the tricuspid valve that was managed for three years without surgical treatment.

## Background

A cardiac mass of the heart is not rare. It is usually found incidentally on routine echocardiography, which provides non-invasive diagnosis of a possible mass with information on the location, attachment, shape, size, and mobility. Echocardiography plays a very important role in the diagnosis of intracardiac masses. Papillary fibroelastoma (PFE) is a benign cardiac tumor that has the potential to cause life-threatening embolic events. Surgical excision of the tumor is recommended for all patients who develop symptoms, but the treatment of asymptomatic patients with an echocardiographically identified PFE is still controversial [1].

## Case presentation

**Figure 1:**
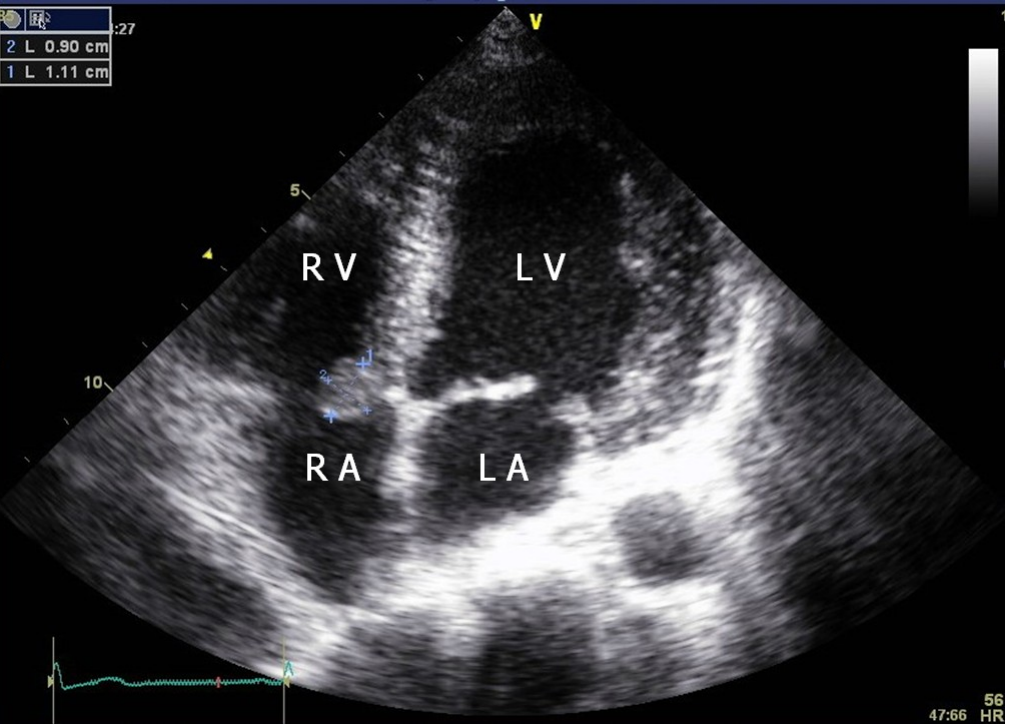
**Modified four-chamber apical view showing an oval (1.1 × 0.9 cm in size) mass having refractive appearance with echolucent areas on the septal leaflet of tricuspid vlave**. LA = left atrium; RA = right atrium; LV = left ventricle; RV = right ventricle.

**Figure 2:**
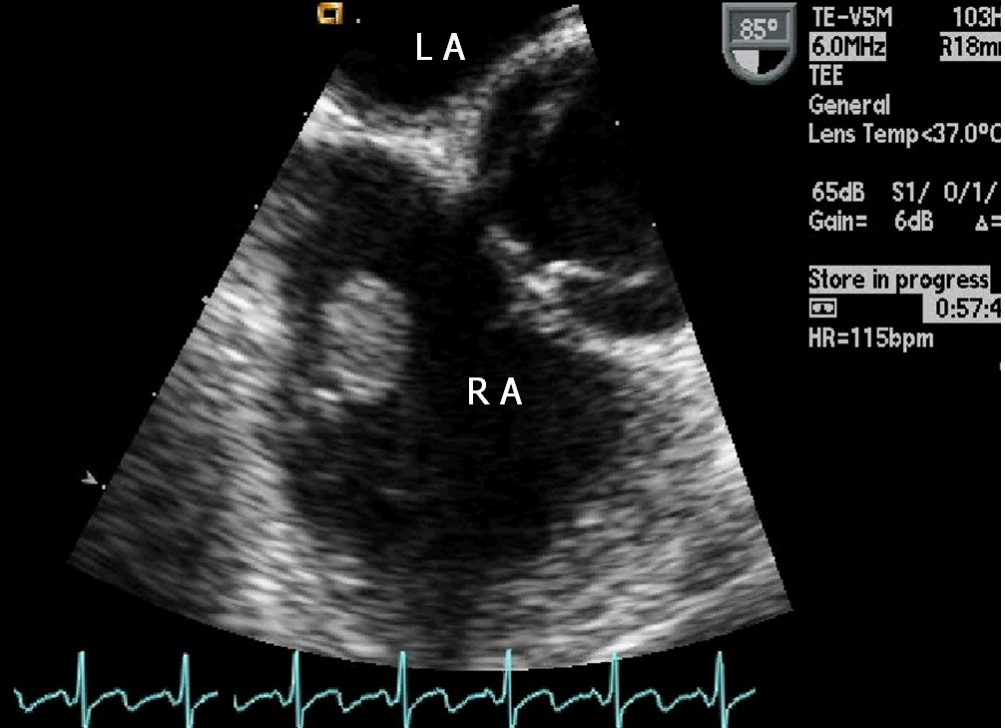
**Transesophageal echocardiography at 85° view showing a mass with a stalk in right atrium**. LA = left atrium; RA = right atrium.

**Figure 3:**
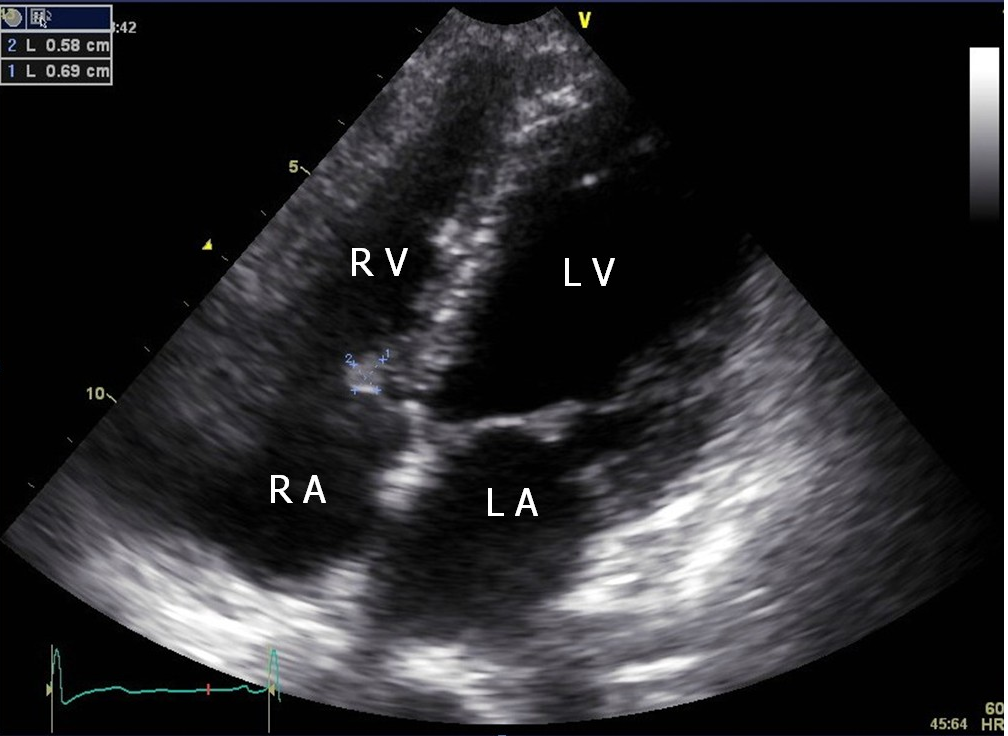
**Three years later, modified four-chamber apical view showing a smaller mass (0.6 × 0.7 cm in size) in comparison with figure 1**. LA = left atrium; RA = right atrium; LV = left ventricle; RV = right ventricle.

A 25-year-old woman was referred from the division of nephrology for evaluation and management of a cardiac mass found on a transthoracic echocardiogram (TTE). The patient had a kidney transplant15 days ago for lupus nephritis. Three years earlier, the echocardiogram revealed mild mitral regurgitation, but no mass was noted on the tricuspid valve and there was no transvalvular blood flow abnormality. Chest X-ray, physical examination, and electrocardiogram were within normal range. Laboratory data, including work-up for infective endocarditis and systemic lupus erythematosus (SLE) were unremarkable except for antinuclear antibodies (ANA) 1:320 (homogenous type) and anti-dsDNA antibodies (+). The ejection fraction was normal and mild mitral regurgitation was noted. The TTE showed an incidental finding of a 1.1 × 0.9 cm mass attached to the septal leaflet of the tricuspid valve (Figure 1) [see Additional file 1]. Transesophageal echocardiography (TEE) revealed a highly mobile, oval mass that had a well-demarcated border with a stalk (Figure 2) [see Additional file 2]. We considered surgical removal of this mass. However, given the patients' co-morbidities and her refusal to undergo surgery, she was followed with conservative treatment, including anticoagulation with heparin for two weeks. However, the mass remained unchanged. The patient was followed at two month intervals for three years after discharge from the hospital. Each follow-up included the evaluation of symptoms. There were no symptoms or complications resulting from the possible embolus. Three years later, the tricuspid mass size was decreased on follow-up echocardiography (Figure 3) [see Additional file 3].

## Discussion

The PFE is the third most common primary tumor of the heart and most frequently involves the cardiac valves [2]. PFEs have well-established echocardiographic characteristics. The sensitivity and specificity of TTE were 88.9% and 87.8%, respectively, with an overall accuracy of 88.4% for the detection of PFE ≥ 0.2 cm [3]. These include a round, oval and irregularly shaped tumor, usually smaller than 1.5 cm, a stalk attachment to the endocardium, they are mobile if attached by such a stalk, and often have a refractive appearance with areas of echo-lucency [3,4]. PFEs are an avascular connective tissue core surrounded by a looser matrix, with multiple adjacent fronds covered by endothelium [3]. These masses must be differentiated from other cardiac mass-like lesions such as myxomas, vegetation, fibromas, Libman-Sacks vegetations, Lamble's excrescences, or thrombi. Myxomas are usually located in the left atrium and originate from the mid portion of the atrium [5]. Infective vegetations are usually associated with clinical signs of endocarditis and valvular destruction, and may resolve or change in appearance over time with treatment [6]. In our patient, the blood tests obtained to rule out endocarditis were negative and specific-endocarditis symptoms were not noted. Fibromas usually occur in children and young adults and typically involve the left ventricle, right ventricle and septum [7]. Lamble's excrescences are considerably smaller and broader-based than PFEs [8]. Thrombi are typically located in the atrial appendage, associated with atrial fibrillation and mitral valve disease or occur in regions with ventricular aneurysm and akinesia, both of which are usually secondary to myocardial infarction or cardiomyopathy and the echocardiographic finding has an irregular or lobulated border and absence of a pedicle [9]. In this patient, It was unlikely to be a thrombus because of the location and features of the mass and the presence of a stalk. Although the patient received anticoagulation treatment with heparin, the mass size remained unchanged. The Libman-Sacks vegetations are generally rounded and sessile, less than 1 cm^2 ^in size, almost exclusively seen on the mitral and aortic valves and rarely on the right heart valves; they have irregular borders, heterogeneous echodensities and no independent motion. Most valves with masses have associated thickening or regurgitation [10].

Most PFEs are asymptomatic; rarely, they are diagnosed because of cardiac symptoms or an embolic event [11]. There are no guidelines for the management of PFEs. No data exist to evaluate the efficacy of anticoagulation or antiplatelet therapy for patients with PFE, although it is speculated that deposition of thrombotic material on the tumors may add to the risk of microembolization [3]. However, as long as there are no definite contraindications to surgery, the only independent predictor of mortality or non-fatal embolization is tumor mobility; surgical excision is strongly recommended for patients with a highly mobile PFE with a stalk [9]. Surgical intervention was the first line treatment recommended for this patient. However, there were no clinical manifestations from the mass such as embolization, followed by heart failure, and the patient refused surgery. And it was relatively the small size mass in the right sided heart. Therefore, the patient was followed closely for three years. There is another case reported with a mobile PFE conservatively managed for more than four years in the literature [1].

## Conclusion

This patient illustrates the nonsurgical management of a pedunculated tricuspid valvular mass, diagnosed by echocardiography, suggesting a PFE. To develop guidelines for standard appropriate management of such patients, randomized controlled trials to compare conservative with surgical management of patients with asymptomatic PFE are needed.

## Consent

Written informed consent was obtained from the patient for publication of this case report and any accompanying images. A copy of the written consent is available for review by the Editor-in-Chief of this journal

## Competing interests

The authors declare that they have no competing interests.

## Authors' contributions

All authors were involved in imaging. SHS drafted the manuscript.

All authors read and approved the final manuscript.

## Supplementary Material

Additional file 1 **Modified apical 4 chamber view**. This is a movie clip, a highly mobile pedunculated mass attached to the septal leaflet of the tricuspid valve. Click here for file

Additional file 2 **Transesophageal view**. This is a movie clip, showing an oval mass. Click here for file

Additional file 3 **Modified apical 4 chamber view**. Another a movie clip, three years later, a smaller mass compared to Figure 1 Click here for file

## References

[B1] Mutlu H, Demir IE, Leppo J, Levy WK (2008). Nonsurgical management of a left ventricular pedunculated papillary fibroelastoma: a case report. J Am Soc Echocardiogr.

[B2] Matsumoto N, Sato Y, Kusama J, Matsuo S, Kinukawa N, Kunimasa T, Ichiyama I, Takahashi H, Kimura S, Orime Y, Saito S (2007). Multiple papillary fibroelastomas of the aortic valve: case report. Int J Cardiol.

[B3] Sun JP, Asher CR, Yang XS, Cheng GG, Scalia GM, Massed AG, Griffin BP, Ratliff NB, Stewart WJ, Yhomas JD (2001). Clinical and echocardiographic characteristics of papillary fibroelastomas: a retrospective and prospective study in 162 patients. Circulation.

[B4] Klarich KW, Enriquez-Sarano M, Gura GM, Edwards WD, Tajik AJ, Seward JB (1997). Papillary fibroelastoma: echocardiographic characteristics for diagnosis and pathologic correlation. J Am Coll Cardiol.

[B5] Reynen K (1995). Cardiac myxomas. N Engl J Med.

[B6] Madu E, Myles J, Fraker TD (1995). Pseudopapillary fibroelastoma of the mitral valve. J Natl Med Assoc.

[B7] Parmley LF, Salley RK, Williams JP, Head GB (1988). The clinical spectrum of cardiac fibroma with diagnostic and surgical considerations: noninvasive imaging enhances management. Ann Thorac Surg.

[B8] Boone SA, Campagna M, Walley VM (1992). Lambl's excrescences and papillary fibroelastomas: are they different?. Can J Cardiol.

[B9] Gowda RM, Khan IA, Nair CK, Mehta NJ, Vasavada BC, Sacchi TJ (2003). Cardiac papillary fibroelastoma: a comprehensive analysis of 725 cases. Am Heart J.

[B10] Gabrielli F, Alcini E, Di Prima MA, Mazzacurati G, Masala C (1995). Cardiac valve involvement in systemic lupus erythematosus and primary antiphospholipid syndrome: lack of correlation with antiphospholipid antibodies. Int J Cardiol.

[B11] Moustafa S, Sauve C, Page P, Serri K (2008). Incidental finding of a papillary fibroelastoma of the mitral valve chordae. Eur J Echocardiogr.

